# A Procedure to Construct Exact Solutions of Nonlinear Fractional Differential Equations

**DOI:** 10.1155/2014/489495

**Published:** 2014-03-10

**Authors:** Özkan Güner, Adem C. Cevikel

**Affiliations:** ^1^Department of Management Information Systems, School of Applied Sciences, Dumlupinar University, 43100 Kutahya, Turkey; ^2^Department of Mathematics Education, Education Faculty, Yildiz Technical University, 34220 Istanbul, Turkey

## Abstract

We use the fractional transformation to convert the nonlinear partial fractional differential equations with the nonlinear ordinary differential equations. The Exp-function method is extended to solve fractional partial differential equations in the sense of the modified Riemann-Liouville derivative. We apply the Exp-function method to the time fractional Sharma-Tasso-Olver equation, the space fractional Burgers equation, and the time fractional fmKdV equation. As a result, we obtain some new exact solutions.

## 1. Introduction

Fractional differential equations (FDEs) are generalizations of classical differential equations of integer order. Recently, fractional differential equations have gained much attention as they are widely used to describe various complex phenomena in various applications such as the fluid flow, signal processing, control theory, systems identification, finance and fractional dynamics, and physics. The fractional differential equations have been investigated by many researchers [1–3]. In recent decades, a large amount of literature has been provided to construct the exact solutions of fractional ordinary differential equations and fractional partial differential equations of physical interest. Many powerful and efficient methods have been proposed to obtain approximate solutions of fractional differential equations, such as the Adomian decomposition method [4, 5], the variational iteration method [6, 7], the homotopy analysis method [8, 9], the homotopy perturbation method [10, 11], and the differential transformation method [12–14]. The fractional subequation method [15–17], the first integral method [18], the Exp-function method [19, 20], and the (G′/G)-expansion method [21–23] can be used to construct the exact solutions for some time and space fractional differential equations.

He and Wu [24] systematically proposed a new method in 2006, called the Exp-function method, to obtain exact solutions of nonlinear differential equations. The Exp-function method has been successfully applied to many kinds of nonlinear differential equations [25–28], such as high-dimensional equations [29–31], variable-coefficient equations [32, 33], differential-difference equations [34, 35], and stochastic equations [36, 37].

The present paper investigates for the first time the applicability and effectiveness of the Exp-function method on fractional nonlinear partial differential equations.

## 2. The Modified Riemann-Liouville Derivative

Jumarie proposed a modified Riemann-Liouville derivative. With this kind of fractional derivative and some useful formulas, we can convert fractional differential equations into integer-order differential equations by variable transformation in [38].

In this section, we firstly give some properties and definitions of the modified Riemann-Liouville derivative which are used further in this paper.

Assume that *f* : *R* → *R*, *x* → *f*(*x*) denote a continuous (but not necessarily differentiable) function. The Jumarie modified Riemann-Liouville derivative of order *α* is defined by the expression

(1) $$\begin{array}{c} D_{x}^{\alpha }f\left(x\right)=\{\begin{array}{cc} \frac{1}{\Gamma \left(-\alpha \right)}\overset{x}{\underset{0}{\int }}{\left(x-\xi \right)}^{-\alpha -1}\left[f\left(\xi \right)-f\left(0\right)\right]d\xi , & \alpha <0,\\ \frac{1}{\Gamma \left(1-\alpha \right)}\frac{d}{dx}\overset{x}{\underset{0}{\int }}{\left(x-\xi \right)}^{-\alpha }\left[f\left(\xi \right)-f\left(0\right)\right]d\xi , & 0<\alpha <1,\\ {\left(f^{\left(n\right)}\left(x\right)\right)}^{\left(\alpha -n\right)}, & n\leq \alpha \leq n+1,\;\;n\geq 1. \end{array} \end{array}$$

A few properties of the fractional modified Riemann-Liouville derivative were summarized and three famous formulas of them are

(2) $$\begin{array}{c} D_{x}^{\alpha }x^{\gamma }=\frac{\Gamma \left(1+\gamma \right)}{\Gamma \left(1+\gamma -\alpha \right)}x^{\gamma -\alpha },\;\gamma >0,\\ D_{x}^{\alpha }\left(u\left(x\right)v\left(x\right)\right)=v\left(x\right)D_{x}^{\alpha }u\left(x\right)+u\left(x\right)D_{x}^{\alpha }v\left(x\right),\\ D_{x}^{\alpha }f\left[u\left(x\right)\right]=f_{u}^{\prime }\left(u\right)D_{x}^{\alpha }u\left(x\right)=D_{u}^{\alpha }f\left(u\right){\left(u_{x}^{\prime }\right)}^{\alpha }, \end{array}$$

which are direct consequences of the equality

(3) $$\begin{array}{c} d^{\alpha }x\left(t\right)=\Gamma \left(1+\alpha \right)dx\left(t\right). \end{array}$$

Secondly, let us consider the time fractional differential equation with independent variables *x* = (*x*_1_, *x*_2_,…, *x*_*m*_, *t*) and a dependent variable *u*:

(4) $$\begin{array}{c} F\left(u,D_{t}^{\alpha }u,u_{x_{1}},u_{x_{2}},u_{x_{3}},D_{t}^{2\alpha }u,u_{x_{1}x_{1}},u_{x_{2}x_{2}},u_{x_{3}x_{3}},\ldots \right)=0. \end{array}$$

Using the fractional variable transformation

(5) $$\begin{array}{c} U\left(\xi \right)=u\left(x_{1},x_{2},\ldots ,x_{m},t\right),\;\\ \xi =x_{1}+l_{1}x_{2}+\cdots +l_{m-1}x_{m}+\frac{\lambda t^{\alpha }}{\Gamma \left(1+\alpha \right)}, \end{array}$$

where *l*_*i*_ and *λ* are constants to be determined later. Similarly, let us consider the space fractional differential equation with independent variables *x* = (*x*_1_, *x*_2_,…, *x*_*m*_, *t*) and a dependent variable *u*:

(6) $$\begin{array}{c} F\left(u,u_{t},D_{x_{1}}^{\beta }u,u_{x_{2}},u_{x_{3}},D_{x_{1}}^{2\beta }u,u_{x_{1}x_{1}},u_{x_{2}x_{2}},u_{x_{3}x_{3}},\ldots \right)=0. \end{array}$$

Next, using the fractional variable transformation

(7) $$\begin{array}{c} U\left(\xi \right)=u\left(x_{1},x_{2},\ldots ,x_{m},t\right),\;\;\;\;\;\;\;\\ \xi =\frac{\lambda x_{1}^{\beta }}{\Gamma \left(1+\beta \right)}+l_{1}x_{2}+\cdots +l_{m-1}x_{m}+l_{m}t, \end{array}$$

where *l*_*i*_ and *λ* are constants to be determined later.

The fractional differential equation (6) is reduced to a nonlinear ordinary differential equation

(8) $$\begin{array}{c} H=\left(U\left(\xi \right),U^{\prime }\left(\xi \right),U^{\prime \prime }\left(\xi \right),\ldots \right), \end{array}$$

where “′” = *d*/*dξ*.

## 3. Description of the Exp-Function Method

We consider the general nonlinear ordinary differential equation in (8). According to Exp-function method, we assume that the wave solution can be expressed in the following form [24]:

(9) $$\begin{array}{c} U\left(\xi \right)=\frac{\sum_{n=-c}^{d}a_{n}\exp\left[n\xi \right]‍\;}{\sum_{m=-p}^{q}b_{m}\exp\left[m\xi \right]‍\;}, \end{array}$$

where *p*, *q*, *c*, and *d* are positive integers which are known to be further determined and *a*_*n*_ and *b*_*m*_ are unknown constants. We can rewrite (9) in the following equivalent form:

(10) $$\begin{array}{c} U\left(\xi \right)=\frac{a_{-c}\exp\left[-c\xi \right]+\cdots +a_{d}\exp\left[d\xi \right]}{b_{-p}\exp\left[-p\xi \right]+\cdots +b_{q}\exp\left[q\xi \right]}. \end{array}$$

This equivalent formulation plays an important and fundamental part for finding the analytic solution of problems. To determine the value of *c* and *p*, we balance the linear term of the highest order of (8) with the highest degree nonlinear term. Similarly, to determine the value of *d* and *q*, we balance the linear term of the lowest order of (8) with the lowest degree nonlinear term.

We suppose that the solution in (8) can be expressed as

(11) $$\begin{array}{c} U\left(\xi \right)=\overset{n}{\underset{i=1}{\sum }}a_{i}\phi^{i}, \end{array}$$

where *ϕ* is the solution of the auxiliary equation *ϕ*′ = *α* + *βϕ* + *γϕ* ^2^. In a similar way, *ϕ* can be expressed in (11).

**Theorem 1**

Suppose that *U* ^(*r*)^ and *U* ^*s*^ are, respectively, the highest order linear term and the highest degree nonlinear term of a nonlinear ODE, where *r* and *s* are both positive integers. Then the balancing procedure using the Exp-function ansatz *U*(*ξ*) = ∑_*n*=−*c*_^*d*^ *a*_*n*_exp⁡(*nξ*)/∑_*m*=−*p*_^*q*^ *b*_*m*_exp⁡(*mξ*) leads to *d* = *q* and *c* = *p* and ∀*r* ≥ 1, ∀*s* ≥ 2 [39].

To show the efficiency of the method described in the previous part, we present some FDEs examples.

## 4. The Time Fractional Sharma-Tasso-Olver Equation

We consider the nonlinear fractional Sharma-Tasso-Olver equation [40]

(12) Dtαu+3aux2+3au2ux+3auuxx+auxxx=0,             t>0, 0<α≤1,

subject to the initial condition

(13) $$\begin{array}{c} u\left(x,0\right)=-\sqrt{2B_{0}}\tan\left(\frac{\sqrt{2B_{0}}}{2}x\right), \end{array}$$

where *a* and *B*_0_ are arbitrary constants and *α* is a parameter describing the order of the fractional time derivative. The function *u*(*x*, *t*) is assumed to be a causal function of time.

For our purpose, we introduce the following transformations:

(14) $$\begin{array}{c} u\left(x,t\right)=U\left(\xi \right),\;\;\xi =x-\frac{\lambda t^{\alpha }}{\Gamma \left(1+\alpha \right)}, \end{array}$$

where *λ* is a constant.

Substituting (14) into (12), we can know that (12) reduced into an ODE

(15) $$\begin{array}{c} -\lambda U^{\prime }+3a{\left(U^{\prime }\right)}^{2}+3aU^{2}U^{\prime }+3aUU^{\prime \prime }+aU^{\prime \prime \prime }=0, \end{array}$$

where “*U*′” = *dU*/*dξ*.

Integrating (15) with respect to *ξ* yields

(16) $$\begin{array}{c} \xi_{0}-\lambda U+3aUU^{\prime }+aU^{3}+aU^{\prime \prime }=0, \end{array}$$

where *ξ*_0_ is a constant of integration.

Here take notice of the nonlinear term in (16), and we can balance *U*′′ and *U* ^3^ by the idea of the Exp-function method [24] to determine the values of *p*, *q*, *c*, and *d*. By simple calculation, we have

(17) $$\begin{array}{c} U^{3}=\frac{c_{1}\exp\left[-\left(3c+p\right)\xi \right]+\cdots }{c_{2}\exp\left[-4p\xi \right]+\cdots },\\ U^{\prime \prime }=\frac{c_{3}\exp\left[-\left(3p+c\right)\xi \right]+\cdots }{c_{4}\exp\left[-4p\xi \right]+\cdots }, \end{array}$$

where *c*_*i*_ are determined coefficients only for simplicity. Balancing the highest order of Exp-function in (17) we have

(18) $$\begin{array}{c} \begin{array}{c} -\left(3p+c\right)=-\left(3c+p\right), \end{array} \end{array}$$

which leads to the result

(19) $$\begin{array}{c} \begin{array}{c} p=c. \end{array} \end{array}$$

Similarly to determine values of *d* and *q*, we balance the linear term of the lowest order in (16):

(20) $$\begin{array}{c} U^{\prime \prime }=\frac{\cdots +d_{1}\exp\left[\left(3q+d\right)\xi \right]}{\cdots +d_{2}\exp\left[4q\xi \right]},\\ \begin{array}{c} U^{3}=\frac{\cdots +d_{3}\exp\left[\left(3d+q\right)\xi \right]}{\cdots +d_{4}\exp\left[4q\xi \right]}, \end{array} \end{array}$$

where *d*_*i*_ are determined coefficients only for simplicity. From (20), we obtain

(21) $$\begin{array}{c} \begin{array}{c} 3q+d=3d+q, \end{array} \end{array}$$

and this gives

(22) $$\begin{array}{c} \begin{array}{c} q=d. \end{array} \end{array}$$

For simplicity, we set *p* = *c* = 1 and *q* = *d* = 1, so (10) reduces to

(23) $$\begin{array}{c} \begin{array}{c} U\left(\xi \right)=\frac{a_{1}\exp\left(\xi \right)+a_{0}+a_{-1}\exp\left(-\xi \right)}{b_{1}\exp\left(\xi \right)+b_{0}+b_{-1}\exp\left(-\xi \right)}. \end{array} \end{array}$$

Substituting (23) into (16) and by the help of symbolic computation, we have

(24) 1A[R3exp⁡⁡(3ξ)+R2exp⁡⁡(2ξ)+R1exp⁡⁡(ξ)+R0  +R−1exp⁡⁡(−ξ)+R−2exp⁡⁡(−2ξ)+R−3exp⁡⁡(−3ξ)] =0,

where

(25) A=(b−1exp⁡⁡(−ξ)+b0+b1exp⁡⁡(ξ))3,R3=−λa1b12+aa13+ξ0b13,R2=aa0b12−λa0b12+3aa12b0+3aa12a0 +3ξ0b12b0−aa1b1b0−2λa1b1b0−3aa1a0b1,R1=−2λa0b1b0+3aa0a1b0−aa0b1b0−3aa02b1 +3aa1a02+aa1b02+3ξ0b1b02−λa1b02 +3ξ0b12b−1−λa−1b12+6aa12b−1 +3aa12a−1+4aa−1b12−2λa1b1b−1 −6aa1a−1b1−4aa1b1b−1,R0=3aa−1b1b0+ξ0b03+aa03−λa0b02 −2λa−1b1b0+9aa1a0b−1−9aa0a−1b1 +6aa1a0a−1+3aa1b0b−1,R−1=−2λa0b−1b0−3aa0a−1b0−aa0b−1b0 −λa−1b02+3aa02b−1+3aa02a−1   +aa−1b02+3ξ0b02b−1+3ξ0b1b−12 −λa1b−12−6aa−12b1+3aa1a−12 +4aa1b−12−2λa−1b1b−1+6aa−1a1b−1 −4aa−1b1b−1,R−2=aa0b−12−λa0b−12−3aa−12b0 +3aa0a−12+3ξ0b0b−12−2λa−1b0b−1 +3aa−1a0b−1−aa−1b0b−1,R−3=−λa−1b−12+ξ0b−13+aa−13.

Solving this system of algebraic equations by using symbolic computation, we obtain the following results.

**Case 1**

We have

(26) $$\begin{array}{c} a_{0}=0,\;\;b_{-1}=-\frac{a_{-1}}{2},\;\;b_{0}=0,\\ b_{1}=\frac{a_{1}}{2},\;\;\xi_{0}=0,\\ \lambda =\lambda ,\;\;a=\frac{\lambda }{4}, \end{array}$$

where *a*_−1_ and *a*_1_ are free parameters. Substituting these results into (23), we obtain the following exact solution:

(27) u(x,t)=(a1exp⁡⁡(x−λtαΓ(1+α))  +a−1exp⁡⁡(−(x−λtαΓ(1+α)))) ×(a12exp⁡⁡(x−λtαΓ(1+α))   −a−12exp⁡⁡(−(x−λtαΓ(1+α))))−1.

If we set *a*_1_ = 2 and *a*_−1_ = −2, (27) becomes

(28) $$\begin{array}{c} u\left(x,t\right)=\tanh\left(x-\frac{\lambda t^{\alpha }}{\Gamma \left(1+\alpha \right)}\right), \end{array}$$

which is the other exact solution of the fractional Sharma-Tasso-Olver equation.

If we set *a*_1_ = *a*_−1_ = 2, (27) becomes

(29) $$\begin{array}{c} u\left(x,t\right)=\coth\left(x-\frac{\lambda t^{\alpha }}{\Gamma \left(1+\alpha \right)}\right), \end{array}$$

which is the other exact solution of the fractional Sharma-Tasso-Olver equation.

**Case 2**

We have

(30) $$\begin{array}{c} a_{0}=0,\;\;b_{-1}=b_{-1},\;\;b_{0}=0,\\ b_{1}=\frac{a_{1}b_{-1}}{\left(a_{-1}+2b_{-1}\right)},\;\;\xi_{0}=\xi_{0},\\ \lambda =\frac{\xi_{0}b_{-1}\left(3a_{-1}^{2}+6a_{-1}b_{-1}+4b_{-1}^{2}\right)}{2a_{-1}\left(a_{-1}+2b_{-1}\right)\left(a_{-1}+b_{-1}\right)},\\ a=\frac{b_{-1}^{3}\xi_{0}}{2a_{-1}\left(a_{-1}+2b_{-1}\right)\left(a_{-1}+b_{-1}\right)}, \end{array}$$

where *a*_−1_ and *b*_−1_ are free parameters. Substituting these results into (23), we obtain the following exact solution:

(31) u(x,t)=(a1exp⁡⁡(x−λtαΓ(1+α))  +a−1exp⁡(−(x−λtαΓ(1+α)))) ×(a1b−1(a−1+2b−1)exp⁡(x−λtαΓ(1+α))   +b−1exp⁡(−(x−λtαΓ(1+α))))−1.

Comparing our results with the results [18, 19], it can be seen that our results are new to our best knowledge.

## 5. The Space Fractional Burgers Equation

We consider the space fractional Burgers equation [41]

(32) ∂u∂t+u∂u∂x−k∂2u∂x2+n∂βu∂xβ=0,     x,t>0, 0<β≤1,

with the following initial value problem:

(33) $$\begin{array}{c} u\left(0,t\right)=0,\;\;u_{x}\left(0,t\right)=\frac{1}{t}-\frac{\pi^{2}}{2kt^{2}}, \end{array}$$

where *k* and *n* are arbitrary constants and *β* is a parameter describing the order of the fractional space derivative. The function *u*(*x*, *t*) is assumed to be a causal function of time.

For our purpose, we introduce the following transformations:

(34) $$\begin{array}{c} u\left(x,t\right)=U\left(\xi \right),\;\;\xi =\frac{\lambda x^{\beta }}{\Gamma \left(1+\beta \right)}-ct, \end{array}$$

where *λ* is a constant.

Substituting (34) into (32), we can know that (32) reduced into an ODE

(35) $$\begin{array}{c} -cU^{\prime }+\lambda UU^{\prime }-k\lambda^{2}U^{\prime \prime }+n\lambda U^{\prime }=0, \end{array}$$

where “*U*′” = *dU*/*dξ*.

Integrating (35) with respect to *ξ* yields

(36) $$\begin{array}{c} \left(\lambda n-c\right)U+\lambda \frac{U^{2}}{2}-k\lambda^{2}U^{\prime }+\xi_{0}=0, \end{array}$$

where *ξ*_0_ is a constant of integration.

Here take notice of the nonlinear term in (36), and we can balance *U*′ and *U* ^2^ by the idea of the Exp-function method [24] to determine the values of *p*, *q*, *c*, and *d*. By simple calculation, we have

(37) $$\begin{array}{c} U^{\prime }=\frac{c_{1}\exp\left[-\left(c+p\right)\xi \right]+\cdots }{c_{2}\exp\left[-2p\xi \right]+\cdots },\\ U^{2}=\frac{c_{3}\exp\left[-2c\xi \right]+\cdots }{c_{4}\exp\left[-2p\xi \right]+\cdots }, \end{array}$$

where *c*_*i*_ are determined coefficients only for simplicity. Balancing the highest order of Exp-function in (37) we have

(38) $$\begin{array}{c} \begin{array}{c} -\left(p+c\right)=-2c, \end{array} \end{array}$$

which leads to the result

(39) $$\begin{array}{c} \begin{array}{c} p=c. \end{array} \end{array}$$

Similarly to determine values of *d* and *q*, we balance the linear term of the lowest order in (36):

(40) $$\begin{array}{c} U^{\prime }=\frac{\cdots +d_{1}\exp\left[\left(q+d\right)\xi \right]}{\cdots +d_{2}\exp\left[2q\xi \right]},\\ \begin{array}{c} U^{2}=\frac{\cdots +d_{3}\exp\left[2d\xi \right]}{\cdots +d_{4}\exp\left[2q\xi \right]}, \end{array} \end{array}$$

where *d*_*i*_ are determined coefficients only for simplicity. From (40), we obtain

(41) $$\begin{array}{c} \begin{array}{c} q+d=2d, \end{array} \end{array}$$

and this gives

(42) $$\begin{array}{c} \begin{array}{c} q=d. \end{array} \end{array}$$

For simplicity, we set *p* = *c* = 1 and *q* = *d* = 1, so (10) reduces to

(43) $$\begin{array}{c} \begin{array}{c} U\left(\xi \right)=\frac{a_{1}\exp\left(\xi \right)+a_{0}+a_{-1}\exp\left(-\xi \right)}{b_{1}\exp\left(\xi \right)+b_{0}+b_{-1}\exp\left(-\xi \right)}. \end{array} \end{array}$$

Substituting (43) into (36) and by the help of computation, we have

(44) 1A[R2exp⁡⁡(2ξ)+R1exp⁡⁡(ξ)+R0  +R−1exp⁡⁡(−ξ)+R−2exp⁡⁡(−2ξ)]=0,

where

(45) A=(b−1exp⁡⁡(−ξ)+b0+b1exp⁡⁡(ξ))2,R2=λ2a12+ξ0b12−ca1b1+nλa1b1,R1=−ca1b0+2ξ0b1b0+λa1a0−ca0b1 +nλa0b1+kλ2a0b1 +nλa1b0−kλ2a1b0,R0=−2kλ2a1b−1+2kλ2a−1b1+nλa1b−1 +nλa0b0+nλb1a−1+12λa02 −ca0b0+ξ0b02+2ξ0b1b−1 −ca1b−1−cb1a−1+λba1a−1,R−1=−ca−1b0−ca0b−1+2ξ0b0b−1 +λa0a−1−kλ2a0b−1+nλa−1b0 +nλa0b−1+kλ2a−1b0,R−2=12λa−12+ξ0b−12−ca−1b−1+nλa−1b−1.

Solving this system of algebraic equations by using symbolic computation, we obtain the following results.

**Case 1**

We have

(46) $$\begin{array}{c} a_{1}=\frac{b_{1}\left(-4k\lambda b_{-1}+a_{-1}\right)}{b_{-1}},\;\;a_{0}=0,\\ k=k,\;\;b_{1}=b_{1},\;\;b_{0}=0,\;\;\lambda =\lambda ,\\ c=\frac{\lambda \left(-2k\lambda b_{-1}+nb_{-1}+a_{-1}\right)}{b_{-1}},\;\;\\ \xi_{0}=\frac{\lambda a_{-1}\left(-4k\lambda b_{-1}+a_{-1}\right)}{2b_{-1}^{2}},\;\;n=n, \end{array}$$

where *a*_−1_, *b*_1_, and *b*_−1_ are free parameters. Substituting these results into (43), we get the following exact solution:

(47) u(x,t)=(b1(−4kλb−1+a−1)b−1exp⁡(λxβΓ(1+β)−ct)  +a−1exp⁡(−(λxβΓ(1+β)−ct))) ×(b1exp⁡⁡(λxβΓ(1+β)−ct)   +b−1exp⁡⁡(−(λxβΓ(1+β)−ct)))−1,

which is the exact solution of the space fractional Burgers equation.

**Case 2**

We have

(48) $$\begin{array}{c} a_{-1}=0,\;\;a_{0}=-\frac{b_{0}\left(\lambda a_{1}+2n\lambda b_{1}-2cb_{1}\right)}{\lambda b_{1}},\\ k=\frac{cb_{1}-\lambda a_{1}-n\lambda b_{1}}{\lambda^{2}b_{1}},\;\;b_{-1}=0,\\ b_{0}=b_{0},\;\;\lambda =\lambda ,\;\;c=c,\\ \xi_{0}=-\frac{a_{1}\left(\lambda a_{1}+2n\lambda b_{1}-2cb_{1}\right)}{2b_{1}^{2}},\;\;n=n, \end{array}$$

where *a*_1_, *b*_0_, and *b*_1_ are free parameters. Substituting these results into (43), we obtain the following exact solution:

(49) u(x,t)=(a1exp⁡(λxβΓ(1+β)−ct)  −b0(λa1+2nλb1−2cb1)λb1) ×(b1exp⁡(λxβΓ(1+β)−ct)+b0)−1,

which is the exact solution of the space fractional Burgers equation.

**Case 3**

We have

(50) a1=−(a−12b02−2a−1b02kλb−1−2a0b0a−1b−1   +2a0b0b−12kλ+a02b−12)(a−1−2kλb−1) ×(4b−14k2λ2)−1,b1=−(a−12b02−2a−1b02kλb−1−2a0b0a−1b−1  +2a0b0b−12kλ+a02b−12)(4b−13k2λ2)−1,a0=a0,  a−1=a−1,  b0=b0,  b−1=b−1,c=λ(a−1−kλb−1+nb−1)b−1,  k=k,  λ=λ,  n=n,  ξ0=λa−1(a−1−2kλb−1)2b−12,

where *a*_0_, *a*_−1_, *b*_0_, and *b*_−1_ are free parameters. Substituting these results into (43), we get the following exact solution:

(51) u(x,t)=(−(a−12b02−2a−1b02kλb−1−2a0b0a−1b−1    +2a0b0b−12kλ+a02b−12)  ×(a−1−2kλb−1)(4b−14k2λ2)−1  ×exp⁡(λxβΓ(1+β)−ct)+a0  +a−1exp⁡(−(λxβΓ(1+β)−ct))) ×(−(a−12b02−2a−1b02kλb−1−2a0b0a−1b−1     +2a0b0b−12kλ+a02b−12)   ×(4b−13k2λ2)−1    ×exp⁡(λxβΓ(1+β)−ct)+b0+b−1     ×exp⁡(−(λxβΓ(1+β)−ct)))−1,

which is the exact solution of the space fractional Burgers equation.

The obtained solutions for the space fractional Burgers equation are new to our best knowledge.

## 6. The Time Fractional fmKdV Equation

We consider the following fractional time fractional fmKdV equation [42]:

(52) Dtαu+u2ux+uxxx=0,  t>0, 0<α≤1,

with the initial conditions as

(53) $$\begin{array}{c} u\left(x,0\right)=\frac{4\sqrt{2}k\sin^{2}\left(kx\right)}{3-\sin^{2}\left(kx\right)}, \end{array}$$

where *k* is an arbitrary constant and *α* is a parameter describing the order of the fractional time derivative.

For our purpose, we introduce the following transformations

(54) $$\begin{array}{c} u\left(x,t\right)=U\left(\xi \right),\;\;\xi =cx-\frac{\lambda t^{\alpha }}{\Gamma \left(1+\alpha \right)}, \end{array}$$

where *λ* and *c* are constants.

Substituting (54) into (52), we can know that (52) reduced into an ODE

(55) $$\begin{array}{c} -\lambda U^{\prime }+cU^{2}U^{\prime }+c^{3}U^{\prime \prime \prime }=0, \end{array}$$

where “*U*′” = *dU*/*dξ*.

By using the ansatz (55), for the linear term of highest order *U*′′′ with the highest order and the nonlinear term *U* ^2^ *U*′, balancing *U*′′′ with *U* ^2^ *U*′ in (55) gives

(56) U′′′=c1exp⁡[−(7p+c)ξ]+⋯c2exp⁡[−8pξ]+⋯=c1exp⁡[−(3p+c)ξ]+⋯c2exp⁡[−4pξ]+⋯,U2U′=c3exp⁡[−(p+3c)ξ]+⋯c4exp⁡[−4pξ]+⋯,

where *c*_*i*_ are determined coefficients only for simplicity. Balancing the highest order of Exp-function in (56) we have

(57) $$\begin{array}{c} \begin{array}{c} -\left(3p+c\right)=-\left(3c+p\right), \end{array} \end{array}$$

which leads to the result

(58) $$\begin{array}{c} \begin{array}{c} p=c. \end{array} \end{array}$$

Similarly to determine values of *d* and *q*, we balance the linear term of the lowest order in (55):

(59) $$\begin{array}{c} U^{\prime \prime \prime }=\frac{\cdots +d_{1}\exp\left[\left(3q+d\right)\xi \right]}{\cdots +d_{2}\exp\left[4q\xi \right]},\\ \begin{array}{c} U^{2}U^{\prime }=\frac{\cdots +d_{3}\exp\left[\left(3d+q\right)\xi \right]}{\cdots +d_{4}\exp\left[4q\xi \right]}, \end{array} \end{array}$$

where *d*_*i*_ are determined coefficients only for simplicity. From (59), we obtain

(60) $$\begin{array}{c} \begin{array}{c} 3q+d=3d+q, \end{array} \end{array}$$

and this gives

(61) $$\begin{array}{c} \begin{array}{c} q=d. \end{array} \end{array}$$

For simplicity, we set *p* = *c* = 1 and *q* = *d* = 1, so (10) reduces to

(62) $$\begin{array}{c} \begin{array}{c} U\left(\xi \right)=\frac{a_{1}\exp\left(\xi \right)+a_{0}+a_{-1}\exp\left(-\xi \right)}{b_{1}\exp\left(\xi \right)+b_{0}+b_{-1}\exp\left(-\xi \right)}. \end{array} \end{array}$$

Substituting (62) into (55) and by the help of computation, we have

(63) 1A[R3exp⁡(3ξ)+R2exp⁡(2ξ)+R1exp⁡(ξ)+R0  +R−1exp⁡⁡(−ξ)+R−2exp⁡⁡(−2ξ)  +R−3exp⁡(−3ξ)]=0,

where

(64) A=(b−1exp⁡⁡(−ξ)+b0+b1exp⁡⁡(ξ))4,R3=λa0b13−c3a0b13+ca13b0 −λa1b12b0−ca12a0b1+c3a1b12b0,R2=2λa−1b13+2ca13b−1−8c3a−1b13 −2ca12a−1b1+8c3a1b12b−1 −2λa1b12b−1−4c3a1b1b02 −2λa1b1b02+2ca12a0b0 −2ca1a02b1+4c3a0b12b0+2λa0b12b0,R1=−λa1b03−ca03b1+c3a1b03 −18c3a1b1b0b−1−6λa1b1b0b−1 −6ca1a0a−1b1+λa0b12b−1 +ca12a−1b0+λa0b1b02 +ca02a1b0−c3a0b1b02 +23c3a0b12b−1+5ca12a0b−1 +5λa−1b12b0−5c3a−1b12b0,R0=4ca12a−1b−1−4ca1a−12b1 −4ca02a−1b1+4c3a1b02b−1 +32c3a−1b12b−1−4c3a−1b1b02 −32c3a1b1b−12−4λa1b1b−12 −4λa1b02b−1+4λa−1b12b−1 +4λa−1b1b02+4ca1a02b−1,R−1=ca03b−1−c3a−1b03+λa−1b03 +6λa−1b1b0b−1+6ca1a−1a0b−1 +18c3a−1b1b0b−1−ca1a−12b0 +c3a0b−1b02−5λa1b0b−12 −ca02a−1b0−5ca0a−12b1 +5c3a1b0b−12−λa0b−1b02 −23c3a0b1b−12−λa0b1b−12,R−2=−2ca0a−12b0−4c3a0b−12b0 +2λa−1b02b−1+2ca02a−1b−1 +2λa−1b1b−12+4c3a−1b02b−1 +2ca−12a1b−1−8c3a−1b1b−12 −2λa0b−12b0+8c3a1b−13 −2λa1b−13−2ca−13b1,R−3=λa−1b0b−12+ca−12a0b−1 −c3a−1b0b−12+c3a0b−13 −ca−13b0−λa0b−13.

Solving this system of algebraic equations by using symbolic computation, we obtain the following results.

**Case 1**

We have

(65) $$\begin{array}{c} a_{0}=a_{0},\;\;a_{1}=-\frac{i\sqrt{6}\left(2a_{0}^{2}+3c^{2}b_{0}^{2}\right)}{24cb_{-1}},\\ a_{-1}=\frac{i\sqrt{6}}{2}cb_{-1},\;\;b_{0}=b_{0},\\ b_{1}=\frac{2a_{0}^{2}+3c^{2}b_{0}^{2}}{12c^{2}b_{-1}},\;\;b_{-1}=b_{-1},\\ \lambda =-\frac{c^{3}}{2}, \end{array}$$

where *a*_0_, *b*_0_, and *b*_−1_ are free parameters. Substituting these results into (62), we obtain the following exact solution:

(66) u(x,t)=(−i6(2a02+3c2b02)24cb−1exp⁡(cx−λtαΓ(1+α))+a0  +(i6/2)cb−1exp⁡(−(cx−λtαΓ(1+α)))) ×(2a02+3c2b0212c2b−1exp⁡(cx−λtαΓ(1+α))+b0+b−1   ×exp⁡(−(cx−λtαΓ(1+α))))−1.

**Case 2**

We have

(67) $$\begin{array}{c} a_{0}=a_{0},\;\;a_{1}=0,\;\;a_{-1}=0,\\ b_{0}=0,\;\;b_{1}=\frac{a_{0}^{2}}{24c^{2}b_{-1}},\\ b_{-1}=b_{-1},\;\;\lambda =c^{3}, \end{array}$$

where *b*_−1_ is a free parameter. Substituting these results into (62), we obtain the following exact solution:

(68) u(x,t)=a0(a0224c2b−1exp⁡(cx−c3tαΓ(1+α))   +b−1exp⁡(−(cx−c3tαΓ(1+α))))−1.

If we set *a*_0_^2^ = 24*c* ^2^ and *b*_−1_ = 1, (68) becomes

(69) $$\begin{array}{c} u\left(x,t\right)=2c\sqrt{6}\text{sech}\left(cx-\frac{c^{3}t^{\alpha }}{\Gamma \left(1+\alpha \right)}\right), \end{array}$$

which is the exact solution of the time fractional fmKdV equation.

**Case 3**

We have

(70) $$\begin{array}{c} a_{0}=\frac{b_{0}\left(a_{-1}^{2}+3c^{2}b_{-1}^{2}\right)}{a_{-1}b_{-1}},\\ a_{1}=\frac{b_{0}^{2}\left(2a_{-1}^{2}+3c^{2}b_{-1}^{2}\right)}{8a_{-1}b_{-1}^{2}},\\ a_{-1}=a_{-1},\\ b_{0}=b_{0},\;\;b_{1}=\frac{b_{0}^{2}\left(2a_{-1}^{2}+3c^{2}b_{-1}^{2}\right)}{8a_{-1}^{2}b_{-1}},\\ b_{-1}=b_{-1},\;\;\lambda =\frac{c\left(a_{-1}^{2}+c^{2}b_{-1}^{2}\right)}{b_{-1}^{2}}, \end{array}$$

where *a*_−1_ and *b*_−1_ are free parameters. Substituting these results into (62), we obtain the following exact solution:

(71) u(x,t)=(b02(2a−12+3c2b−12)8a−1b−12exp⁡(cx−λtαΓ(1+α))   +b0(a−12+3c2b−12)a−1b−1   +a−1exp⁡(−(cx−λtαΓ(1+α)))) ×(b02(2a−12+3c2b−12)8a−12b−1exp⁡(cx−λtαΓ(1+α))   +b0+b−1exp⁡(−(cx−λtαΓ(1+α))))−1.

**Case 4**

We have

(72) $$\begin{array}{c} a_{0}=\frac{i\sqrt{6}}{2}cb_{0},\;\;a_{1}=-\frac{i\sqrt{6}}{2}cb_{1},\\ a_{-1}=0,\;\;b_{0}=b_{0},\;\;b_{1}=b_{1},\\ b_{-1}=0,\;\;\lambda =-\frac{c^{3}}{2}, \end{array}$$

where *b*_0_ and *b*_1_ are free parameters. Substituting these results into (62), we obtain the following exact solution:

(73) u(x,t)  =i6c2(b1exp⁡(cx+(c3/2)tα/Γ(1+α))−b0b1exp⁡(cx+(c3/2)tα/Γ(1+α))+b0).

If we set *b*_0_ = 1 and *b*_1_ = 1, (73) becomes

(74) u(x,t)=i6c2(sinh⁡⁡(cx+(c3/2)tαΓ(1+α))     +cosh⁡(cx+(c3/2)tαΓ(1+α))−1) ×(sinh⁡⁡(cx+(c3/2)tαΓ(1+α))   +cosh⁡⁡(cx+(c3/2)tαΓ(1+α))+1)−1,

which is the exact solution of the time fractional fmKdV equation.

**Case 5**

We have

(75) $$\begin{array}{c} a_{0}=0,\;\;a_{1}=-i\sqrt{6}cb_{1},\;\;a_{-1}=i\sqrt{6}cb_{-1},\\ b_{0}=0,\;\;b_{1}=b_{1},\;\;b_{-1}=b_{-1},\\ \lambda =-2c^{3}, \end{array}$$

where *b*_1_ and *b*_−1_ are free parameters. Substituting these results into (62), we have the following exact solution:

(76) u(x,t)=(−i6cb1exp⁡(x+2c3tαΓ(1+α))  +i6cb−1exp⁡(−(x+2c3tαΓ(1+α)))) ×(b1exp⁡(x+2c3tαΓ(1+α))   +b−1exp⁡(−(x+2c3tαΓ(1+α))))−1.

If we take *b*_1_ = *b*_−1_ = 1, (76) becomes

(77) $$\begin{array}{c} u\left(x,t\right)=-ic\sqrt{6}\tanh\left(x+\frac{2c^{3}t^{\alpha }}{\Gamma \left(1+\alpha \right)}\right), \end{array}$$

which is the exact solution of the time fractional fmKdV equation.

The established solutions have been checked by putting them back into the original equation (52). To the best of our knowledge, they have not been obtained in literature.

## 7. Conclusion

In this paper, we use the Exp-function method to calculate the exact solutions for the time and space fractional nonlinear partial differential equations. When the parameters take certain values, the solitary wave solutions are derived from the exponential form. Since this method is very efficient, reliable, simple, and powerful in finding the exact solutions for the nonlinear fractional differential equations, the proposed method can be extended to solve many systems of nonlinear fractional partial differential equations. We hope that the present solutions may be useful in further numerical analysis and these results are going to be very useful in further future research.
